# Study of Antibodies to Cytolethal Distending Toxin B (CdtB) and Antibodies to Vinculin in Patients with Irritable Bowel Syndrome

**DOI:** 10.12688/f1000research.52086.1

**Published:** 2021-04-19

**Authors:** Maysaa El Sayed Zaki, Dina Elhammady, Mona Foda Salama, Mostafa Abdelsalam, Asmaa Osama Bakr Osman

**Affiliations:** 1 Clinical Pathology, Mansoura University, Mansoura, Egypt; 2 Tropical Medicine, Mansoura University, Mansoura, Egypt; 3 Medical Microbiology and Immunology, Mansoura University, Mansoura, Egypt; 4 Nephrology and Dialysis Unit, Internal Medicine Department,, Mansoura University, Mansoura, Egypt; 5 Clinical Pathology, Assuit University, Assuit, Egypt

**Keywords:** irritable bowel syndrome, anti-vinculin, anti-CdtB, Rome IV

## Abstract

**Background: **Irritable bowel syndrome (IBS) is a common gastrointestinal disorder, categorized into various subtypes. Post-infection IBS may be attributed to the release of cytolethal distending toxin B (CdtB), which cross-reacts with the adhesion protein vinculin responsible for normal intestinal contractility.

**Objective:** This study aims to identify anti-CdtB and anti-vinculin levels in IBS patients compared to healthy control.

**Subjects and methods: **This retrospective case-control study was conducted on 100 patients with IBS, as determined by a questionnaire based on Rome IV criteria, recruited from the outpatient clinics of the Tropical Medicine at Mansoura University Hospital from January 2019 to January 2020.

**Results:** Anti-vinculin and anti-CdtB levels were significantly elevated in patients with IBS (1.58±0.496ng/ml, 2.47±0.60ng/ml)  when compared to control subjects (1.13±0.249ng/ml, 2.1±0.24 ng/ml), respectively with P=0.001 for both.  Anti-vinculin level was significantly higher in the IBS-D subtype than the other subtypes (P=0.001) while, Anti-CdtB was significantly elevated in IBS-C, IBS-D subgroups compared to control subjects (P=0.001).

**Conclusion:** Findings of the present study support the hypothesis that IBS results from post-infectious disorders initiated by bacterial enteritis. A hypothesis could be applied to all IBS subgroups. On the other hand. These biomarkers might reflect the post-infectious state's severity. These findings need further extensive longitudinal studies in patients with IBS.

## Introduction

Irritable bowel syndrome (IBS) is a common gut disorder that affects approximately 11% of the global population. ^1, 2^ IBS mainly manifests in abdominal pain with bowel habit changes in the absence of either radiological evidence of associated pathological conditions or detectable chemical and physiological abnormalities. The diagnosis of this clinical condition relies upon Rome criteria. ^3– 7^

To understand the pathogenesis of irritable bowel syndrome (IBS), previous studies have developed a rat model utilizing infection with *Campylobacter jejuni* in order to elicit a post-infection phenotype resembling human post-infection IBS (PI-IBS) characterized by apparent changes in the composition of small intestinal microbiota. ^8, 9^ In these studies, progression to IBS was accompanied by the detection of a specific bacterial toxin named cytolethal distending toxin B (CdtB), a potential factor attributing to the pathogenesis of PI-IBS. This was supported by the low incidence of IBS in patients infected with a mutant strain of *C. jejuni* that lacks CdtB. ^8, 10^

Furthermore, the development of antibodies to CdtB was associated with altering gut microbiota associated with reducing specific interstitial cells of Cajal (ICC). ^11, 12^ These findings are possibly due to the ability of anti-CdtB to cross-react with the host cell adhesion protein vinculin present in interstitial cells of Cajal and the myenteric ganglia that control the normal activity of the intestinal tract, including phase III of inter-digestive motor activity. ^13^ Absence or decrease in phase III contractions results in small intestinal bacterial overgrowth in animal models and human patients with IBS. ^14, 15^ In this sense, autoimmunity may profoundly affect the host immune response to infections with *C. jejuni*, subsequently leading to IBS. ^16, 17^ Based on these data, it has been suggested that loss of vinculin in the neuromuscular system of the GIT may be associated with the affection of the gut in animal models of post-infection *C. jejuni.* Detection of circulating levels of anti-CdtB and anti-vinculin by enzyme-linked immunosorbent assay (ELISA) has been used to identify patients with IBS-D, ^18^ and to differentiate it from other IBS subtypes. ^19^

The present study aims to detect anti-CdtB and anti-vinculin levels in patients with IBS and their possible role in the diagnosis of different IBS subtypes.

## Methods

This was a prospective case-control study comprising 100 adult patients aged >18 years with IBS, recruited from the Tropical Medicine Department's outpatient clinics at Mansoura University Hospital from January 2019 to January 2020, in addition to 100 healthy individuals as a control group. According to the actual calculated sample size, we needed to enroll 385 with IBS and 193 for control group at a power of 80% and type I error = 0.05, while at a power of 99% and type I error = 0.01, the minimum number of the patients was 16585 cirrhotic patients and 830 for the control group. This is very hard to achieve in lower economy countries like Egypt. We cannot afford to measure all the parameters in these patients. We had to design the study depending on self-funding without any further support.

### Selection and exclusion criteria

Patients were recruited and determined by a questionnaire-based upon the Rome IV criteria, then classified according to their predominant stool composition over 25% of the time: into IBS-C (hard or lumpy stools), IBS-D (loose and watery stools), or IBS-M (a mix of both types). ^19^ Exclusion criteria included patients with hepatic, renal, or autoimmune diseases, those with history of inflammatory bowel disease, gastrointestinal surgeries, thyroid disorders, diabetes mellitus, and patients with a history of taking antibiotics in the last 30 days.

### Laboratory methods

A 10 ml blood sample was obtained from each subject, which was then divided into three aliquots. Two aliquots were used to determine complete blood counts, and one aliquot was utilized for serum separation to assess complete liver function tests, including alanine transaminase, aspartate transaminase, total bilirubin, total albumin, and the kidney function test creatinine. The third aliquot was overlaid on heparin for plasma separation, and the remaining sera were stored at -20°C to be used for evaluation of anti-vinculin antibodies by laboratory prepared ELISA and anti-CdtB antibodies by commercial ELISA (Creative Diagnostics. 45-16 Ramsey Road Shirley, NY 11967, USA).

### ELISA for anti-vinculin

Anti-vinculin levels were measured in separated plasma using human vinculin protein in a concentration of 1.21.2 μg/ml (Novoprotein Scientific, Summit, New Jersey, USA) as an antigen. The vinculin was used to coat wells of the plate following overnight incubation in the wells at 4°C with 100 mmol/l borate buffered saline AQ4 at a pH of 8.2 (Sigma-Aldrich). The reaction was blocked by using BSA 3% and incubating for one hour at room temperature, then washing three times with 0.05% PBS and Tween 20 (pH 7.4). Plasma was added after a 1:32 dilution in saline, then antibodies for vinculin (R and D Systems Cat# MAB6896, RRID:AB_10992930), were added as positive control and incubated for one hour at room temperature followed by washing three times with 0.05% PBS and Tween 20 (pH 7.4). Horseradish peroxidase-conjugated secondary antibodies (Millipore–Merck) were added and incubated for one hour at room temperature. After washing, a tetramethylbenzidine substrate solution (BioRad) was used for detection using a micro-plate reader (stat Fax-1200; Awareness Technology, Florida, USA). Optical densities (ODs) were read at 370nm, and the results were interpreted as OD. ^12^

### ELISA for anti-CdtB (creative diagnostics)

The kit applies the double-sandwich technique using a pre-coated microplate with anti-CDTB antigen. Samples and antigen were added into ELISA plate wells and washed out with PBS, after which Avidin-peroxidase conjugates were added to ELISA wells in order. Tetramethyl benzidine (TMB) substrate was used for coloring after PBS thoroughly washed out the reactant. TMB turns blue in peroxidase reaction and finally turns yellow under the action of acid. Optical densities (ODs) were read at 450, and the results were interpreted as ng/ml.

## Statistical analysis

Data are reported as means and standard deviation (SD) or counts and percentages when appropriate. Comparisons between groups were made using t-tests, Mann-Whitney tests, Chi-square, or Fisher exact tests dictated by data type and distribution.

One-way analysis of variance (ANOVA) was used to test differences between more than two groups. P-value < 0.05 was considered significant for all statistical analyses in this study. All analyses were performed using the Statistical Package of Social Sciences (SPSS) version 22 for Windows (SPSS, Inc., Chicago, IL, USA).

## Results

**Figure 1.**
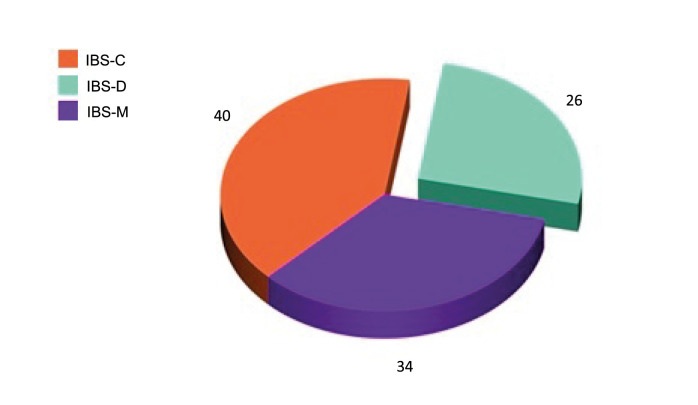
Distribution of patients according to Rome III criteria.

This study included 100 patients with IBS (49 males and 51 females) aged 46.6 ± 6.8 years and 100 healthy controls with a statistically insignificant difference between patients and control regarding age and sex ( *P* = 0.8 and *P* = 0.6, respectively). Patients were classified according to Rome III criteria into 40 patients with IBS-C, 26 patients with IBS-D, and 34 patients with IBS-M ( Figure 1). Laboratory investigations, including ALT, AST, albumin, total bilirubin, hemoglobin, total leucocytes count, platelets, and creatinine, showed non-significant differences between patients and control subjects ( *P* = 0.6, *P* = 0.5, *P* = 0.7, *P* = 0.6, *P* = 0.99, *P* = 0.99, and *P* = 0.58) respectively ( Table 1).

**Table 1.** Comparison of demographic and laboratory findings between patients and control subjects.

Parameter	Patients with IBS (n = 100)	Healthy Control (n = 100)	*P*
**Age** *(Mean ± SD)*	50.1 ± 6.6	46.6 ± 6.8	0.8
**Sex** • **Male** *(No/%)* • **Female** *(No/%)*	49 (49%) 51 (51%)	49 (49%) 51 (51%)	0.6
**Hemoglobin** *(Mean ± SD) gm/dl*	13.19 ± 1.7	13.16 ± 1.7	0.99
**Total leucocytes count** *(Mean ± SD) × 10 ^3^/mm ^3^*	13.2 ± 1.7	13.1 ± 1.8	0.99
**Platelets** *(Mean ± SD) × 10 ^3^/mm ^3^*	134.15 ± 56.32	141.35 ± 34.04	0.003
**Creatinine** *(Mean ± SD) mg/dl*	0.98 ± 0.25	0.96 ± 0.28	0.58
**ALT** *(Mean ± SD) IU/l*	28.85 ± 4.7	29.2 ± 4.4	0.6
**AST** *(Mean ± SD)*	27.26 ± 4.32	27.7 ± 4.05	0.5
**Albumin** *(Mean ± SD) gm/dl*	4.00 ± 0.51	4.1 ± 0.52	0.7
**Total bilirubin** *(Mean ± SD) mg/dl*	0.82 ± 0.10	0.9 ± 0.11	0.6

Anti-vinculin and anti-CdtB levels were significantly elevated in IBS patients (1.58 ± 0.496ng/ml and 2.47 ± 0.60 ng/ml) respectively compared to the control subjects (1.13 ± 0.249 ng/ml and 2.1 ± 0.24 ng/ml) respectively with *P* = 0.001 for both ( Table 2).

**Table 2.** Comparison of anti-vinculin and anti-CdtB in patients with IBS versus control subjects.

Parameter	Patients with IBS *(Mean ± SD)*	Control Subjects *(Mean ± SD)*	*P*
**Anti-vinculin **	1.58 ± 0.496	1.13 ± 0.249	0.001
**Anti-CdtB **	2.47 ± 0.60	2.1 ± 0.24	0.001

Anti-vinculin levels were also significantly higher in different IBS subgroups compared to control subjects, with the anti-vinculin level being significantly elevated in the IBS-D subtype when compared to the other subtypes with *P* = 0.001. Similarly, anti-CdtB showed significant elevation in IBS-C and IBS-D compared to control subjects ( *P* = 0.001), with a significantly higher level detected in IBS-D than IBS-C ( *P* = 0.001). However, the level of anti-CdtB in IBS-M was detected at a non-significant lower level compared to control subjects ( *P* = 0.2), but at a significantly lower level when compared to IBS-C and IBS-D ( *P* = 0.001) ( Table 3).

**Table 3.** Comparison of anti-vinculin and anti-CdtB between different subgroups of IBS and control subjects.

Parameter	IBS-C (N = 40) *(Mean ± SD)*	IBS-D (N = 26) *(Mean ± SD)*	IBS-M (N = 34) *(Mean ± SD)*	Control (N = 100) *(Mean ± SD)*	*P*
**Anti-vinculin **	1.33 ± 0.49	1.84 ± 0.42	1.68 ± 0.42	1.13 ± 0.25	P1 = 0.001 P2 = 0.001 P3 = 0.001 P4 = 0.01 P5 = 0.001 P6 = 0.001
**Anti-CdtB **	2.52 ± 0.46	2.98 ± 0.6	2.03 ± 0.67	2.1 ± 0.24	P = 0.001 P1 = 0.001 P2 = 0.001 P3 = 0.001 P4 = 0.2 P5 = 0.001 P6 = 0.001

. P1 comparing IBS-C and IBS-D. . P2 comparing IBS-C and IBS-M. . P3 comparing IBS-D and IBS-M. . P4 comparing IBS-M and control subjects. . P5 comparing IBS-IBS-C and control subjects. . P6 comparing IBS-D and control subjects.

## Discussion

There is an extreme necessity for the utilization of accessible and reliable, low-cost biomarkers in identifying IBS for the low-risk population to reduce the use of colonoscopy. ^20^ Previous studies have recognized anti-CdtB and anti-vinculin for use as valuable biomarkers in different IBS patients from healthy controls ^21, 22^ in different populations. However, these biomarkers have not been sufficiently evaluated in Egyptian patients.

In the current study, both anti-vinculin and anti-CdtB demonstrated significantly elevated levels in IBS patients when compared to the control subjects, a finding that mirrors those from a previous study by Talley et al. ^23^ However, data reported by Rezaie et al. ^16^ depicts significant elevation in levels of both biomarkers only in IBS-M and IBS-D, but not IBS-C. This discrepancy in findings may be attributed to the difference in etiology of different IBS subtypes, ^4^ as it is hypothesized that most cases of post-infectious IBS manifest as IBS-D or IBS-M, with a minority of patients manifesting as IBS-C. ^9^ Another factor may make the microbiome profile difference between IBS patient subgroups; bacterial species producing methane are decreased in IBS-D and IBS-M ^23^ and increased in IBS-C. ^24^ Patients included in the present study, particularly those in the IBS-C subgroup, may represent patients who develop IBS following infections associated with their microbiota profile changes. These findings need extensive longitudinal studies to be confirmed.

Anti-vinculin and anti-CdtB levels in this study were significantly elevated in patients with IBS-D, a concordance finding with Pimentel et al., ^21^ who reported that anti-CdtB and anti-vinculin distinguished IBS-D from IBD or other organic GI disease and healthy control. On the other hand, Bayoumy et al. ^25^ reported that anti-vinculin could be an important biomarker for IBS-D diagnosis among Egyptian patients. Cytolethal distending toxin represents a virulence factor for bacterial pathogens such as *Escherichia coli, Salmonella, Shigella*, and *Campylobacter jejuni*, by causing epithelial barrier breakdown and suppression of the acquired immune response to invading pathogens, resulting in an amplified pro-inflammatory response with consequent persistence of bacterial infection. ^16^ Development of anti-CdtB antibodies occurs in response to secretion of cytolethal distending toxin following infection with bacterial pathogens. Molecular mimicry accounts for the potential cross-reaction between anti-CdtB and vinculin with resultant anti-vinculin autoantibody production leading to injury to interstitial cells of Cajal (ICC) with the development of IBS. ^12^ Based on the suggestion of an association between the metabolic syndrome and liver affection and IBS, this study group performed liver function tests as a simple evaluation of liver affection. However, liver enzymes were normal in IBS patients' studied group, in contrast to reports by Lee et al. ^26^

The principal limitation of the present study was the small sample size. This necessitates the extension of the study to include large sample size. The findings of the present study are supported by previous studies (18, 19).

## Conclusion

The present study’s findings support the hypothesis that IBS results from post-infectious disorders initiated by bacterial enteritis. Moreover, this hypothesis can be applied to all IBS subgroups as both anti-CdtB and anti-vinculin biomarkers were significantly elevated in IBS-C and IBS-D subgroups, with only anti-vinculin being elevated in IBS-M when compared to healthy control. These biomarkers were significantly elevated in IBS-D compared to IBS-C and IBS-M, possibly reflecting the post-infectious state's severity. These findings need further extensive longitudinal studies in patients with IBS.

## Consent

All participants provided written informed consent and the study was conducted according to the principles outlined in the Declaration of Helsinki. Confidentiality and privacy were considered regarding personal, clinical and laboratory data.

## Ethical approval

Mansoura Faculty of Medicine Institutional Research Board approved the research (R.21.01.1141).

## Data availability

Figshare: “Study of antibodies to cytolethal distending toxin B (CdtB) and antibodies to vinculin in patients with irritable bowel syndrome” https://doi.org/10.6084/m9.figshare.14178908.v1. ^27^

Data are available under the terms of the Creative Commons CC BY 4.0

## References

[1] Lacy BE Patel NK : Rome Criteria and a Diagnostic Approach to Irritable Bowel Syndrome. *J Clin Med.* 2017;6(11):99. 10.3390/jcm6110099 29072609 PMC5704116

[2] Canavan C West J Card T : The epidemiology of irritable bowel syndrome. *Clin Epidemiol.* 2014;6:71–80. 10.2147/CLEP.S40245 24523597 PMC3921083

[3] Longstreth GF Thompson WG Chey WD : Functional bowel disorders. *Gastroenterology.* 2006;130(5):1480–1491. 10.1053/j.gastro.2005.11.061 16678561

[4] Tibble JA Sigthorsson G Foster R : Use of surrogate markers of inflammation and Rome criteria to distinguish organic from nonorganic intestinal disease. *Gastroenterology.* 2002;123:450–460. 10.1053/gast.2002.34755 12145798

[5] Longstreth GF Thompson WG Chey WD : Functional bowel disorders. *Gastroenterology.* 2006;130:1480–1491. 10.1053/j.gastro.2005.11.061 16678561

[6] Schmulson MJ Drossman DA : What is new in Rome IV. *J Neurogastroenterol Motil.* 2017 Apr 30;23(2):151–63. 10.5056/jnm16214 28274109 PMC5383110

[7] Ford AC Bercik P Morgan DG : Validation of the Rome III criteria for the diagnosis of irritable bowel syndrome in secondary care. *Gastroenterology.* 2013;145:1262–1270. 10.1053/j.gastro.2013.08.048 23994201

[8] Jee SR Morales W Low K : ICC density predicts bacterial overgrowth in a rat model of post-infectious IBS. *World J Gastroenterol.* 2010;16:3680–3686. 10.3748/wjg.v16.i29.3680 20677340 PMC2915428

[9] Morales W Pimentel M Hwang L : Acute and chronic histological changes of the small bowel secondary to C. jejuni infection in a rat model for post-infectious IBS. *Dig Dis Sci.* 2011;56:2575–2584. 10.1007/s10620-011-1662-6 21409374

[10] Pokkunuri V Pimentel M Morales W : Role of Cytolethal Distending Toxin in Altered Stool Form and Bowel Phenotypes in a Rat Model of Post-infectious Irritable Bowel Syndrome. *J Neurogastroenterol Motil.* 2012;18:434–442. 10.5056/jnm.2012.18.4.434 23106005 PMC3479258

[11] Morales W Weitsman S Kim G : Circulating antibodies to cytolethal distending toxin B correlate with the development of small intestinal bacterial overgrowth in a rat model of post-infectious IBS. *Gastroenterology.* 2013;144:S-931–932.

[12] Pimentel M Morales W Pokkunuri V : Autoimmunity Links Vinculin to the Pathophysiology of Chronic Functional Bowel Changes Following Campylobacter jejuni Infection in a Rat Model. *Dig Dis Sci.* 2015 May;60(5):1195–205. 10.1007/s10620-014-3435-5 25424202

[13] Vanderwinden JM Liu H De Laet MH : Study of the interstitial cells of Cajal in infantile hypertrophic pyloricstenosis. *Gastroenterology.* 1996;111:279–288. 10.1053/gast.1996.v111.pm8690192 8690192

[14] Nieuwenhuijs VB Verheem A van Duijvenbode-Beumer H : The role of interdigestive small bowel motility in the regulation of gut microflora, bacterial overgrowth, and bacterial translocation in rats. *Ann Surg.* 1998;228:188–193. 10.1097/00000658-199808000-00007 9712563 PMC1191459

[15] Vantrappen G Janssens J Hellemans J : The interdigestivemotor complex of normal subjects and patients with bacterial overgrowth of the small intestine. *J Clin Invest.* 1977;59:1158–1166. 10.1172/JCI108740 864008 PMC372329

[16] Rezaie A Park SC Morales W : Assessment of anti-vinculin and anti-cytolethal distending toxin B antibodies in subtypes of irritable bowel syndrome. *Dig Dis Sci.* 2017;62:1480–1485. 10.1007/s10620-017-4585-z 28451914

[17] Lombardero M Heymann PW Platts-Mills TA : Conformational stability of B cell epitopes on group I and group II *Dermatophagoides* spp. allergens. Effect of thermal and chemical denaturation on the binding of murine IgG and humanIgE antibodies. *J Immunol.* 1990;144:1353–1360. 1689351

[18] Morales W Rezaie A Barlow G : Second-Generation Biomarker Testing for Irritable Bowel Syndrome Using Plasma Anti-CdtB and Anti-Vinculin Levels. *Dig Dis Sci.* 2019 Nov;64(11):3115–3121. 10.1007/s10620-019-05684-6 31152332

[19] Rezaie A Park SC Morales W : Assessment of Anti-vinculin and Anti-cytolethal Distending Toxin B Antibodies in Subtypes of Irritable Bowel Syndrome. *Dig Dis Sci.* 2017 Jun;62(6):1480–1485. 10.1007/s10620-017-4585-z 28451914

[20] Burbige EJ : Irritable bowel syndrome: Diagnostic approaches in clinical practice. *Clin Exp Gastroenterol.* 2010;3:127. 10.2147/CEG.S12596 21694856 PMC3108663

[21] Pimentel M Morales W Rezaie A : Development and validation of a biomarker for diarrhea-predominant irritable bowel syndrome in human subjects. *PLoS One.* 2015;10(5):e0126438. 10.1371/journal.pone.0126438 25970536 PMC4430499

[22] Talley NJ Holtmann G Walker MM : Circulating Anti-cytolethal Distending Toxin B and Anti-vinculin Antibodies as Biomarkers in Community and Healthcare Populations With Functional Dyspepsia and Irritable Bowel Syndrome. *Clin Transl Gastroenterol.* 2019;10(7):e00064. 10.14309/ctg.0000000000000064 31356481 PMC6708662

[23] Pozuelo M Panda S Santiago A : Reduction of butyrate- and methane-producing microorganisms in patients with Irritable Bowel Syndrome. *Sci Rep.* 2015;5:12693. 10.1038/srep12693 26239401 PMC4523847

[24] Kim G Deepinder F Morales W : Methanobrevibacter smithii is the predominant methanogen in patients with constipation-predominant IBS and methane on breath. *Dig Dis Sci.* 2012;57:3213–3218. 10.1007/s10620-012-2197-1 22573345

[25] Bayoumi E Sabrya M Soliman NRA : Antivinculin antibodies as a marker of irritable bowel syndrome–diarrhea in Egyptian patients. *Egyp Liver J.* 2018;8(4):132–135.

[26] Lee SH Kim KN Kim KM : Irritable Bowel Syndrome May Be Associated with Elevated Alanine Aminotransferase and Metabolic Syndrome. *Yonsei Med J.* 2016;57(1):146–152. 10.3349/ymj.2016.57.1.146 26632395 PMC4696946

[27] Zaki EM Elhammady D Abdelsalam M : Study of antibodies to cytolethal distending toxin B (CdtB) and antibodies to vinculin in patients with irritable bowel syndrome. *figshare. Dataset.* 2021. 10.6084/m9.figshare.14178908.v1 PMC8546732 34754418

