# Supplementary material for: Study of Antibodies to Cytolethal Distending Toxin B (CdtB) and Antibodies to Vinculin in Patients with Irritable Bowel Syndrome
Source: F1000Res. 2021 Oct 15;10:303. Originally published 2021 Apr 19. [Version 4] doi: 10.12688/f1000research.52086.4 (PMC8546732; doi:10.12688/f1000research.52086.4)
Supplement: Peer review history

# Peer review: Reviewer response for version 4

**DOI:** 10.5256/f1000research.77874.r97035

**Published:** 2021-10-25

**Authors:** Gabriela Leite

**Affiliations:** 1 Medically Associated Science and Technology (MAST) Program, Cedars-Sinai Medical Center, Los Angeles, CA, USA

Minor change:

- Authors did not correct the cdtB and vinculin units in the results section as requested previously. - "The OD of the anti-vinculin and anti-CdtB levels were significantly elevated in IBS patients (1.58 ± 0.496ng/ml and 2.47 ± 0.60 ng/ml) respectively compared to the control subjects (1.13 ± 0.249 ng/ml and 2.1 ± 0.24 ng/ml) respectively with *P* = 0.001 for both ( Table 2)."

Is the work clearly and accurately presented and does it cite the current literature?

Partly

If applicable, is the statistical analysis and its interpretation appropriate?

Yes

Are all the source data underlying the results available to ensure full reproducibility?

Partly

Is the study design appropriate and is the work technically sound?

Yes

Are the conclusions drawn adequately supported by the results?

Partly

Are sufficient details of methods and analysis provided to allow replication by others?

Yes

Reviewer Expertise:

Drug development, test development for diagnosis of antibodies and antigens associated with bacterial diseases.

I confirm that I have read this submission and believe that I have an appropriate level of expertise to confirm that it is of an acceptable scientific standard.

# Peer review: Reviewer response for version 3

**DOI:** 10.5256/f1000research.58688.r94996

**Published:** 2021-09-29

**Authors:** Simon Keely

**Affiliations:** 1 College of Health, Medicine and Wellbeing, University of Newcastle, Callaghan, NSW, Australia

I have no further comments.

Is the work clearly and accurately presented and does it cite the current literature?

Partly

If applicable, is the statistical analysis and its interpretation appropriate?

Partly

Are all the source data underlying the results available to ensure full reproducibility?

Yes

Is the study design appropriate and is the work technically sound?

Yes

Are the conclusions drawn adequately supported by the results?

Yes

Are sufficient details of methods and analysis provided to allow replication by others?

Yes

Reviewer Expertise:

Gastroenterology, mucosal inflammation, immunology, microbiome, functional GI disorders.

I confirm that I have read this submission and believe that I have an appropriate level of expertise to confirm that it is of an acceptable scientific standard.

# Response

**Published:** 2021-09-29

**Authors:** Maysaa El Zaki

**Affiliations:** Mansoura University, Egypt

Thanks Sir for your great efforts

# Peer review: Reviewer response for version 3

**DOI:** 10.5256/f1000research.58688.r92547

**Published:** 2021-08-31

**Authors:** Gabriela Leite

**Affiliations:** 1 Medically Associated Science and Technology (MAST) Program, Cedars-Sinai Medical Center, Los Angeles, CA, USA

The authors did not correct the units for antibodies anti-vinculin and anti-cdtB as suggested previously. Neither Results or Abstract were corrected on version 3 of the manuscript. The authors need to clarify the choice of reporting the values as ng/mL, considering that analysis were performed with OD values as stated in Methods.

Is the work clearly and accurately presented and does it cite the current literature?

Partly

If applicable, is the statistical analysis and its interpretation appropriate?

Yes

Are all the source data underlying the results available to ensure full reproducibility?

Partly

Is the study design appropriate and is the work technically sound?

Yes

Are the conclusions drawn adequately supported by the results?

Partly

Are sufficient details of methods and analysis provided to allow replication by others?

Yes

Reviewer Expertise:

Drug development, test development for diagnosis of antibodies and antigens associated with bacterial diseases.

I confirm that I have read this submission and believe that I have an appropriate level of expertise to confirm that it is of an acceptable scientific standard, however I have significant reservations, as outlined above.

# Response

**Published:** 2021-09-01

**Authors:** Maysaa El Zaki

**Affiliations:** Mansoura University, Egypt

The results of units for antibodies anti-vinculin and anti-cdtB was expressed as OD values as mentioned in the method section. I will add the OD to the abstract and in the result section.

# Response

**Published:** 2021-09-21

**Authors:** Maysaa El Zaki

**Affiliations:** Mansoura University, Egypt

The OD was added to all sections of the study as it was the correct used unit.

# Peer review: Reviewer response for version 2

**DOI:** 10.5256/f1000research.57371.r88732

**Published:** 2021-07-09

**Authors:** Gabriela Leite

**Affiliations:** 1 Medically Associated Science and Technology (MAST) Program, Cedars-Sinai Medical Center, Los Angeles, CA, USA

The authors addressed and clarified all comments. Thank you for that. One last adjustment:

 On the paragraph below from Results, the units for cdtB and vinculin antibodies levels should be double checked. The levels were measured considering the OD results. If authors included a standard curve on the test, the details should be included in Methods. If no standard curve was included, the test can not be reported as ng/mL Thank you.

 "Anti-vinculin and anti-CdtB levels were significantly elevated in IBS patients (1.58 ± 0.496ng/ml and 2.47 ± 0.60 ng/ml) respectively compared to the control subjects (1.13 ± 0.249 ng/ml and 2.1 ± 0.24 ng/ml) respectively with *P* = 0.001 for both ( Table 2).Anti-vinculin and anti-CdtB levels were significantly elevated in IBS patients (1.58 ± 0.496ng/ml and 2.47 ± 0.60 ng/ml) respectively compared to the control subjects (1.13 ± 0.249 ng/ml and 2.1 ± 0.24 ng/ml) respectively with *P* = 0.001 for both ( Table 2)."

Is the work clearly and accurately presented and does it cite the current literature?

Partly

If applicable, is the statistical analysis and its interpretation appropriate?

Yes

Are all the source data underlying the results available to ensure full reproducibility?

Partly

Is the study design appropriate and is the work technically sound?

Yes

Are the conclusions drawn adequately supported by the results?

Partly

Are sufficient details of methods and analysis provided to allow replication by others?

Yes

Reviewer Expertise:

Drug development, test development for diagnosis of antibodies and antigens associated with bacterial diseases.

I confirm that I have read this submission and believe that I have an appropriate level of expertise to confirm that it is of an acceptable scientific standard, however I have significant reservations, as outlined above.

# Response

**Published:** 2021-07-13

**Authors:** Maysaa El Zaki

**Affiliations:** Mansoura University, Egypt

Thanks for your accurate review, I will respond

# Response

**Published:** 2021-07-13

**Authors:** Maysaa El Zaki

**Affiliations:** Mansoura University, Egypt

- *On the paragraph below from Results, the units for cdtB and vinculin antibodies levels should be double checked. The levels were measured considering the OD results. If authors included a standard curve on the test, the details should be included in Methods. If no standard curve was included, the test can not be reported as ng/mL Thank you.*
- **Response: **Corrected

# Peer review: Reviewer response for version 2

**DOI:** 10.5256/f1000research.57371.r88731

**Published:** 2021-07-07

**Authors:** Simon Keely

**Affiliations:** 1 College of Health, Medicine and Wellbeing, University of Newcastle, Callaghan, NSW, Australia

It is very disappointing to see the author's responses to my comments. The comments have largely been dismissed, ignored or superficially addressed and there are errors and discrepancies in the article that have not been amended.

 Referring to my previous comments:

1. The response is satisfactory.
2. The authors amended text "the following was added: 'In the present study there was no history of previous infection with *C. jejuni*. However, the elevated levels of antiCdtB and antivinculin can be used as biomarkers for diagnosis of IBS either post infections or without previous infection" is not supported by these results. The authors do not know if the patients were previously infected and thus there is no evidence to suggest that the test can detect or discern post-infectious and idiopathic IBS. The authors have no way of confirming the individuals in their cohort are truly without previous infection. If anything, the data suggest that PI IBS is more common than is realised.
3. The authors still refer to the Rome IV criteria in their Abstract and Methods (and keyword). But their results (paragraph 1) figure legends (Figure 1) suggest that they have classified patients on Rome III criteria. The fact that the authors ignored or misunderstood this critique calls into question the validity of the methodology.
4. The authors have removed the sample size calculation, largely in line with the second reviewer's suggestion. However, without the sample size calculation the conclusion "The principal limitation of the present study was the small sample size. This necessitates the extension of the study to include large sample size." is unfounded. How can you conclude you are underpowered if you have not provided a sample size calculation?
5. The authors choose not to discuss this limitation.
6. With larger studies published and no sample size, the conclusion that a larger study is required is not valid and should be removed.

Is the work clearly and accurately presented and does it cite the current literature?

Partly

If applicable, is the statistical analysis and its interpretation appropriate?

Partly

Are all the source data underlying the results available to ensure full reproducibility?

Yes

Is the study design appropriate and is the work technically sound?

Yes

Are the conclusions drawn adequately supported by the results?

Yes

Are sufficient details of methods and analysis provided to allow replication by others?

Yes

Reviewer Expertise:

Gastroenterology, mucosal inflammation, immunology, microbiome, functional GI disorders.

I confirm that I have read this submission and believe that I have an appropriate level of expertise to confirm that it is of an acceptable scientific standard, however I have significant reservations, as outlined above.

# Response

**Published:** 2021-07-07

**Authors:** Maysaa El Zaki

**Affiliations:** Mansoura University, Egypt

The response will be performed in more detail.

# Response

**Published:** 2021-07-13

**Authors:** Maysaa El Zaki

**Affiliations:** Mansoura University, Egypt

- *The authors amended text "the following was added: 'In the present study there was no history of previous infection with C. jejuni. However, the elevated levels of antiCdtB and antivinculin can be used as biomarkers for diagnosis of IBS either post infections or without previous infection" is not supported by these results. The authors do not know if the patients were previously infected and thus there is no evidence to suggest that the test can detect or discern post-infectious and idiopathic IBS. The authors have no way of confirming the individuals in their cohort are truly without previous infection. If anything, the data suggest that PI IBS is more common than is realised.*
- **Response: **The following was added: 'The data of the present study support that PI IBS may be more common than it is realized'.  
- *The authors still refer to the Rome IV criteria in their Abstract and Methods (and keyword). But their results (paragraph 1) figure legends (Figure 1) suggest that they have classified patients on Rome III criteria. The fact that the authors ignored or misunderstood this critique calls into question the validity of the methodology.*
- **Response: **Corrected
- *The authors have removed the sample size calculation, largely in line with the second reviewer's suggestion. However, without the sample size calculation the conclusion "The principal limitation of the present study was the small sample size. This necessitates the extension of the study to include large sample size." is unfounded. How can you conclude you are underpowered if you have not provided a sample size calculation?*
- **Response: **The recommendation of larger sample size was removed
- *Were any additional data taken on patients that may impact results, BMI or anxiety/depression, for instance? There are previous studies that suggest that HADS can improve the sensitivity of blood based biomarkers for FGIDs (Jones et al. (2014)).*
- **Response: **Added as a limitation as there was no psychological studies performed for those patients
- *With larger studies published and no sample size, the conclusion that a larger study is required is not valid and should be removed.*
- **Response:** Removed

# Peer review: Reviewer response for version 1

**DOI:** 10.5256/f1000research.55313.r85381

**Published:** 2021-06-11

**Authors:** Simon Keely

**Affiliations:** 1 College of Health, Medicine and Wellbeing, University of Newcastle, Callaghan, NSW, Australia

This is an interesting study validating previous work on the utility of CdtB and vinculin as potential biomarkers for functional GI disorders, specifically IBS.

 The study is clearly reported although there are a number of inconsistencies that need to be addressed.

 There are also a number of statements that would benefit from revision with the more current literature in mind.

1. The introduction speculates on microbiome alterations in IBS however it should be noted that the idea of a specific IBS microbiome is someone controversial with larger studies analysing mucosal microbiomes showing now distinct signature (Hugerth *et al*. (2021 ^1^)).
2. The introduction discusses the association between CdtB and vinculin antibodies and post-infectious IBS and *C. jejuni*, however there is no mention of whether the patients have a post infectious history (reading the manuscript, one assumes they are idiopathic IBS cases) and the increases in these antibodies in a potentially non PI cohort is not addressed in the discussion. This warrants revision.
3. Methodology - the abstract and methods state that these are Rome IV diagnosed patients but the results state that they are Rome III. Please explain this discrepancy.
4. The power calculation is very concerning. The line " the minimum number of the patients was 16585 cirrhotic patients and 830 for the control group" appears to be copied from a different power calculation for a different study. There should be no reason to do this as the power calculation should be independent. Please show the detailed power calculation for this study, including the data used to estimate the sample size.
5. Were any additional data taken on patients that may impact results, BMI or anxiety/depression, for instance? There are previous studies that suggest that HADS can improve the sensitivity of blood based biomarkers for FGIDs (Jones *et al.* (2014 ^2^)).
6. The conclusion is that a larger sample cohort is warranted, but this study validates previous, larger studies. 

Is the work clearly and accurately presented and does it cite the current literature?

Partly

If applicable, is the statistical analysis and its interpretation appropriate?

Partly

Are all the source data underlying the results available to ensure full reproducibility?

Yes

Is the study design appropriate and is the work technically sound?

Yes

Are the conclusions drawn adequately supported by the results?

Yes

Are sufficient details of methods and analysis provided to allow replication by others?

Yes

Reviewer Expertise:

Gastroenterology, mucosal inflammation, immunology, microbiome, functional GI disorders.

I confirm that I have read this submission and believe that I have an appropriate level of expertise to confirm that it is of an acceptable scientific standard, however I have significant reservations, as outlined above.

## References

[1] : No distinct microbiome signature of irritable bowel syndrome found in a Swedish random population. *Gut* .69(6) : 10.1136/gutjnl-2019-318717 1076-1084 10.1136/gutjnl-2019-318717 31601615PMC7282555

[2] : A biomarker panel and psychological morbidity differentiates the irritable bowel syndrome from health and provides novel pathophysiological leads. *Aliment Pharmacol Ther* .2014;39(4) : 10.1111/apt.12608 426-37 10.1111/apt.12608 24387672

# Response

**Published:** 2021-06-11

**Authors:** Maysaa El Zaki

**Affiliations:** Mansoura University, Egypt

Thanks for reviewing the article. The response will be followed.

# Response

**Published:** 2021-06-15

**Authors:** Maysaa El Zaki

**Affiliations:** Mansoura University, Egypt

**Response to reviewers:**

 We would like to thank the reviewer the insightful comments on the paper, as these comments led us to an improvement of the work. Our revisions reflect all reviewers suggestions. Detailed responses to the reviewers are given below.

- *This is an interesting study validating previous work on the utility of CdtB and vinculin as potential biomarkers for functional GI disorders, specifically IBS.*
- *The study is clearly reported although there are a number of inconsistencies that need to be addressed.*
- *There are also a number of statements that would benefit from revision with the more current literature in mind.*
- **Response: **Thanks, we really appreciate that.
- *The introduction speculates on microbiome alterations in IBS however it should be noted that the idea of a specific IBS microbiome is someone controversial with larger studies analysing mucosal microbiomes showing now distinct signature (Hugerth et al. (2021)).*
- **Response:** Thanks for your nice comment, we mentioned this observation in the introduction.
- *The introduction discusses the association between CdtB and vinculin antibodies and post-infectious IBS and C. jejuni, however there is no mention of whether the patients have a post infectious history (reading the manuscript, one assumes they are idiopathic IBS cases) and the increases in these antibodies in a potentially non PI cohort is not addressed in the discussion. This warrants revision.*
- **Response:** The following was added: 'In the present study there was no history of previous infection with *C. jejuni.* However, the elevated levels of antiCdtB and antivinculin can be used as biomarkers for diagnosis of IBS either post infections or without previous infection'.
- *Methodology - the abstract and methods state that these are Rome IV diagnosed patients but the results state that they are Rome III. Please explain this discrepancy.*
- **Response:** Thanks for your meticulous observation but we depend on Rome IV and we divided patients according to the results of the questionnaire into three groups as there were no patients unclassified.
- *The power calculation is very concerning. The line " the minimum number of the patients was 16585 cirrhotic patients and 830 for the control group" appears to be copied from a different power calculation for a different study. There should be no reason to do this as the power calculation should be independent. Please show the detailed power calculation for this study, including the data used to estimate the sample size.*
- **Response:** Thanks for your comments, it was corrected. 
- *Were any additional data taken on patients that may impact results, BMI or anxiety/depression, for instance? There are previous studies that suggest that HADS can improve the sensitivity of blood based biomarkers for FGIDs (Jones et al. (2014)).*
- **Response**: Thanks for your observation but actually we did not have full data about these points.

 

- *The conclusion is that a larger sample cohort is warranted, but this study validates previous, larger studies. *
- **Response: **Thanks, we agree with you our study validates previous larger studies but to the best of our knowledge this was the first study which discussed this point in Egypt. Unfortunately, due to lack of financial support it was difficult to do the study on large scale of patients. 

# Peer review: Reviewer response for version 1

**DOI:** 10.5256/f1000research.55313.r86122

**Published:** 2021-05-27

**Authors:** Gabriela Leite

**Affiliations:** 1 Medically Associated Science and Technology (MAST) Program, Cedars-Sinai Medical Center, Los Angeles, CA, USA

In this study, Zaki and collaborators observed increased levels of plasma CdtB and vinculin antibodies in IBS subjects when compared to controls (non-IBS subjects) and analysis also revealed differences between IBS subtypes. The study has an important clinical relevance, but to be published, several corrections and clarifications should be addressed. The major concern is about the cdtB antibody test. It is not clear which kit was used for this test, authors need to clarify and double-check if the clostridium difficile toxin B (CDTB) kit was used. CdtB from *C. difficile* is a binary toxin termed the *C. difficile* toxin (CDT) and it has pore-forming or delivery subunit termed CDTb. This toxin is not the same of that observed in *Campylobacter jejuni*, the cytolethal distending toxin B. These are two completely different toxins. All publications with cdtB in IBS subjects were based on cdtB from *C. jejuni*. I couldn’t find the ELISA kit the authors mentioned on Creative Diagnostics webpage.

*Suggestions of writing are marked in italic.*

**Abstract**

- Authors need to restructure the abstract considering suggestions on the manuscript.
- Overall suggestion: the word “patients” should be replaced by “subjects”.

 

**Introduction**

 Paragraph 01:

- “IBS mainly manifests in *subjects with *abdominal pain and bowel habit changes in the absence…”
- In between Paragraph 01 and 02, authors should present info about IBS subtypes.

 

 Paragraph 02:

- “Irritable bowel syndrome” needs to be substituted by IBS.
-  “In these studies, progression to IBS was accompanied by the detection of *circulating levels *of a specific bacterial toxin named cytolethal distending toxin B (CdtB), a potential factor attributing to the pathogenesis of PI-IBS.”
- “This was supported by the low incidence of IBS in patients infected with a mutant strain of *C. jejuni *that lacks CdtB.” These studies were based on results in rats, not patients.

 

 Paragraph 03:

- “These findings *were linked *to the ability of anti-CdtB to cross-react *with vinculin, a host cell adhesion protein *present in interstitial cells of Cajal and the myenteric ganglia that control the normal activity of the intestinal tract, including phase III of inter-digestive motor activity.”
- “Based on these data, it has been suggested that loss of vinculin in the neuromuscular system of the GIT may be associated with the affection of the gut in animal models of post-infection *C. jejuni*.” This statement needs a reference. GIT needs to be spelled out.

Paragraph 04”

- “The present study aims to detect *and to quantify *anti-CdtB and anti-vinculin levels *in subjects *with IBS and their possible role in the diagnosis of different IBS subtypes.”

 

**Methods**

- In the abstract, authors mentioned this study was based in a retrospective study, but in Methods, a prospective study was mentioned
- The authors provided information about samples size calculation (which in my opinion is not needed for this study), but authors mentioned the need of 16585 cirrhotic patients (Paragraph 01 in Methods). Not sure why cirrhotic subjects.
- “According to the actual calculated sample size, we needed to enroll 385 with IBS and 193 for control group at a power of 80% and type I error = 0.05, while at a power of 99% and type I error = 0.01, the minimum number of the patients was 16585 cirrhotic patients and 830 for the control group. This is very hard to achieve in lower economy countries like Egypt. We cannot afford to measure all the parameters in these patients. We had to design the study depending on self-funding without any further support.” I don’t think this info is needed. Since this is a retrospective study, 100 subjects with matched control group (gender and age) should give you enough power to check differences in quantities/levels. If you are planning to define a cutoff for clinical/diagnosis purpose, then you would need a sample size calculation.
- “Patients were recruited, *and IBS was *determined by a questionnaire-based upon the Rome IV criteria”
- “The third aliquot was overlaid on heparin for plasma separation, and the remaining sera were stored at -20°C to be used for evaluation of anti-vinculin antibodies by laboratory prepared ELISA and anti-CdtB antibodies by commercial ELISA (Creative Diagnostics. 45-16 Ramsey Road Shirley, NY 11967, USA).” If heparin was used on third aliquot, everything should be plasma, not serum. What was the kit catalog number used for this assay? I only could find the kit “Human Anti-Clostridium Difficile Toxin B (CDTB) ELISA” on Creative Diagnostics webpage. CdtB from this kit refers to *clostridium difficile* toxin B, but cdtB from IBS studies refers to cytolethal distending toxin B, mostly from *C. jejuni*. This information should be clarified as a major priority. The toxin B from *Clostridium* is not the same as cdtB from *C. jejuni.*   
- “Anti-vinculin levels were measured in separated plasma using human vinculin protein in a concentration of 1.21.2 μg/ml (Novoprotein Scientific, Summit, New Jersey, USA) as an antigen.” Vinculin concentration should be fixed.
- Authors said that “Optical densities (ODs) were read at 370nm, and the results were interpreted as OD”, but vinculin antibodies were reported as ng/mL. Please explain. If a calculation was performed, this need to be described.

 

**Results**

- “This study included 100 patients with IBS (49 males and 51 females) aged 46.6 ± 6.8 years and 100 healthy controls *(please add sex distribution and mean age/SD here). No differences were observed between IBS subjects and control *regarding age and sex (P = 0.8 and P = 0.6, respectively).” I added a suggestion.
- “Patients were classified according to Rome III criteria into 40 patients with IBS-C, 26 patients with IBS-D, and 34 patients with IBS-M (Figure 1).” Authors mentioned Rome IV criteria in Methods. Figure 1 is not needed.
- “Anti-vinculin and anti-CdtB levels were significantly elevated in IBS patients (1.58 ± 0.496 ng/ml and 2.47 ± 0.60 ng/ml) respectively compared to the control subjects (1.13 ± 0.249 ng/ml and 2.1 ± 0.24 ng/ml) respectively with P = 0.001 for both (Table 2).” Authors need to double check vinculin antibodies unit.
- In table 3, the first P-value of the anti-cdtB results was calculated based on what? All the other P-values (P1 to P6) are explained on the text below the table.

 

**Discussion**

- Overall suggestion: the discussion can be better expanded.
- “There is an extreme necessity for the utilization of accessible and reliable, low-cost biomarkers in identifying IBS for the low-risk population to reduce the use of colonoscopy”. Low-risk population? What do the authors mean with low risk? Please expand a little bit more why the need of development of a test for IBS.
- “Previous studies have recognized anti-CdtB and anti-vinculin for use as valuable biomarkers in different IBS patients from healthy controls”. Please rephrase it. It is confusing. I encourage the authors to perform a language review of the manuscript. Some suggestions were already included in my review, but I strongly recommend the authors to perform a in deep language review of the entire manuscript.  
- “This discrepancy in findings may be attributed to the difference in etiology of different IBS subtypes, as it is hypothesized that most cases of post-infectious IBS manifest as IBS-D or IBS-M, with a minority of patients manifesting as IBS-C.  Another factor may make the microbiome profile difference between IBS patient subgroups; bacterial species producing methane are decreased in IBS-D and IBS-M and increased in IBS-C. Patients included in the present study, particularly those in the IBS-C subgroup, may represent patients who develop IBS following infections associated with their microbiota profile changes. These findings need extensive longitudinal studies to be confirmed.” These statements are very confusing, not sure if that can be used to explain why IBS-C also had increased cdtB and vinculin antibodies. This needs to be better clarified. In addition, methane producers are mostly Archaea, not bacteria. Increases in methanogens also represents microbiome changes.
- “Anti-vinculin and anti-CdtB levels in this study were significantly elevated in patients with IBS-D, a concordance finding with Pimentel *et al.*,  who reported that anti-CdtB and anti-vinculin distinguished IBS-D *from IBD, other *organic GI *diseases *and healthy control. *In addition,* Bayoumy *et al.*  reported that anti-vinculin could be an important biomarker for IBS-D diagnosis among Egyptian patients.”
- “ Cytolethal distending toxin *(use abbreviation) is* a virulence factor for bacterial pathogens such as *Escherichia coli, Salmonella, Shigella*, and *Campylobacter jejuni*, by causing epithelial barrier breakdown and suppression of the acquired immune response to invading pathogens, resulting in an amplified pro-inflammatory response with consequent persistence of bacterial infection. Development of anti-CdtB antibodies occurs in response to secretion of cytolethal distending toxin *(use abbreviation) *following infection with bacterial pathogens. Molecular mimicry accounts for the potential cross-reaction between anti-CdtB and vinculin with resultant anti-vinculin autoantibody production leading to injury to interstitial cells of Cajal (ICC) with the development of IBS. Based on the suggestion of an association between the metabolic syndrome and liver affection and IBS, this study group performed liver function tests as a simple evaluation of liver affection. However, liver enzymes were normal in IBS patients' studied group, in contrast to reports by Lee *et al*.” This entire paragraph should be rewritten, it is confusing and not well constructed.

 

**Conclusion**

- “The *present findings *support the hypothesis that IBS *may *results from *post-infectious bacterial gastroenteritis. *Moreover, this hypothesis can be applied to all IBS subgroups as both anti-CdtB and anti-vinculin biomarkers were significantly elevated in IBS-C and IBS-D subgroups, with only anti-vinculin being elevated in IBS-M when compared to healthy control.”
- “These biomarkers were significantly elevated in IBS-D compared to IBS-C and IBS-M, possibly reflecting the post-infectious state's severity. These findings need further extensive longitudinal studies in patients with IBS.” How was severity of the disease accessed? There is no mention of that in any part of the manuscript, so no conclusion can be made based on severity.

Is the work clearly and accurately presented and does it cite the current literature?

Partly

If applicable, is the statistical analysis and its interpretation appropriate?

Yes

Are all the source data underlying the results available to ensure full reproducibility?

Partly

Is the study design appropriate and is the work technically sound?

Yes

Are the conclusions drawn adequately supported by the results?

Partly

Are sufficient details of methods and analysis provided to allow replication by others?

Yes

Reviewer Expertise:

Drug development, test development for diagnosis of antibodies and antigens associated with bacterial diseases.

I confirm that I have read this submission and believe that I have an appropriate level of expertise to confirm that it is of an acceptable scientific standard, however I have significant reservations, as outlined above.

# Response

**Published:** 2021-05-30

**Authors:** Maysaa El Zaki

**Affiliations:** Mansoura University, Egypt

**Response to reviewers:**

 We would like to thank the reviewer the insightful comments on the paper, as these comments led us to an improvement of the work. Our revisions reflect all reviewers suggestions. Detailed responses to the reviewers are given below.

- *In this study, Zaki and collaborators observed increased levels of plasma CdtB and vinculin antibodies in IBS subjects when compared to controls (non-IBS subjects) and analysis also revealed differences between IBS subtypes. The study has an important clinical relevance, but to be published, several corrections and clarifications should be addressed.*
- **Response**: Many thanks, we really appreciate that.
- *The major concern is about the cdtB antibody test. It is not clear which kit was used for this test, authors need to clarify and double-check if the clostridium difficile toxin B (CDTB) kit was used. CdtB from C. difficile is a binary toxin termed the C. difficile toxin (CDT) and it has pore-forming or delivery subunit termed CDTb. This toxin is not the same of that observed in Campylobacter jejuni, the cytolethal distending toxin B. These are two completely different toxins. All publications with cdtB in IBS subjects were based on cdtB from C. jejuni. I couldn’t find the ELISA kit the authors mentioned on Creative Diagnostics webpage*.
- **Response:** Thanks for your meticulous comment and observation and we already clarify this point in the methodology as the measured was anticdtB from C.jejuni.

**Abstract**

- *Authors need to restructure the abstract considering suggestions on the manuscript. Overall suggestion: the word “patients” should be replaced by “subjects”.*
- **Response: **Thanks, we corrected it.

**Introduction**

 Paragraph 01:

- *“IBS mainly manifests in subjects with abdominal pain and bowel habit changes in the absence…”*
- **Response: **Thanks, corrected.
- *In between Paragraph 01 and 02, authors should present info about IBS subtypes.*
- **Response: **Thanks for your suggestion, we added it.

Paragraph 02:

- *“Irritable bowel syndrome” needs to be substituted by IBS.*
- **Response:** Thanks, corrected.

- *“In these studies, progression to IBS was accompanied by the detection of circulating levels of a specific bacterial toxin named cytolethal distending toxin B (CdtB), a potential factor attributing to the pathogenesis of PI-IBS.”*
- **Response: **Thanks for your advise, corrected.

- *“This was supported by the low incidence of IBS in patients infected with a mutant strain of C. jejuni that lacks CdtB.” These studies were based on results in rats, not patients.*
- **Response:** Thanks for meticulous observation, modified.

Paragraph 03:

- *“These findings were linked to the ability of anti-CdtB to cross-react with vinculin, a host cell adhesion protein present in interstitial cells of Cajal and the myenteric ganglia that control the normal activity of the intestinal tract, including phase III of inter-digestive motor activity.”*
- **Response**: Thanks, corrected.
- *“Based on these data, it has been suggested that loss of vinculin in the neuromuscular system of the GIT may be associated with the affection of the gut in animal models of post-infection C. jejuni.” This statement needs a reference. GIT needs to be spelled out.*
- **Response: **Thanks, corrected.

Paragraph 04:

- *“The present study aims to detect and to quantify anti-CdtB and anti-vinculin levels in subjects with IBS and their possible role in the diagnosis of different IBS subtypes.”*
- **Response:** **Thanks, corrected.

**Methods**

- *In the abstract, authors mentioned this study was based in a retrospective study, but in Methods, a prospective study was mentione*d
- **Response: **Thanks for your comment, corrected
- The authors provided information about samples size calculation (which in my opinion is not needed for this study), but authors mentioned the need of 16585 cirrhotic patients (Paragraph 01 in Methods). Not sure why cirrhotic subjects.
- *“According to the actual calculated sample size, we needed to enroll 385 with IBS and 193 for control group at a power of 80% and type I error = 0.05, while at a power of 99% and type I error = 0.01, the minimum number of the patients was 16585 cirrhotic patients and 830 for the control group. This is very hard to achieve in lower economy countries like Egypt. We cannot afford to measure all the parameters in these patients. We had to design the study depending on self-funding without any further support.” I don’t think this info is needed. Since this is a retrospective study, 100 subjects with matched control group (gender and age) should give you enough power to check differences in quantities/levels. If you are planning to define a cutoff for clinical/diagnosis purpose, then you would need a sample size calculation*
- **Response:** Thanks for your advise and suggestion. This calculation was added as a response to the editor of the journal and now it is removed as your suggestion.
- *“Patients were recruited, and IBS was determined by a questionnaire-based upon the Rome IV criteria”*
- **Response: **Thanks, corrected.
- *“The third aliquot was overlaid on heparin for plasma separation, and the remaining sera were stored at -20°C to be used for evaluation of anti-vinculin antibodies by laboratory prepared ELISA and anti-CdtB antibodies by commercial ELISA (Creative Diagnostics. 45-16 Ramsey Road Shirley, NY 11967, USA).” If heparin was used on third aliquot, everything should be plasma, not serum. What was the kit catalog number used for this assay? I only could find the kit “Human Anti-Clostridium Difficile Toxin B (CDTB) ELISA” on Creative Diagnostics webpage. CdtB from this kit refers to clostridium difficile toxin B, but cdtB from IBS studies refers to cytolethal distending toxin B, mostly from C. jejuni. This information should be clarified as a major priority. The toxin B from Clostridium is not the same as cdtB from C. jejuni. * 
- **Response:** Thanks for your comments, the study of antibodies for anti cdtB was performed on serum samples as clarified in the methods and the commercial refers to the used components of the ELISA method used. The plasma was used for anti-vinculin antibodies.

- *“Anti-vinculin levels were measured in separated plasma using human vinculin protein in a concentration of 1.21.2 μg/ml (Novoprotein Scientific, Summit, New Jersey, USA) as an antigen.” Vinculin concentration should be fixed.*
- **Response**: Thanks for your observation, corrected.

- *Authors said that “Optical densities (ODs) were read at 370nm, and the results were interpreted as OD”, but vinculin antibodies were reported as ng/mL. Please explain. If a calculation was performed, this need to be described.*
- **Response:** Thanks for your insightful advise, we corrected it.
- *“This study included 100 patients with IBS (49 males and 51 females) aged 46.6 ± 6.8 years and 100 healthy controls (please add sex distribution and mean age/SD here). No differences were observed between IBS subjects and control regarding age and sex (P = 0.8 and P = 0.6, respectively).” I added a suggestion.*
- **Response**: Thanks, corrected.
- *“Patients were classified according to Rome III criteria into 40 patients with IBS-C, 26 patients with IBS-D, and 34 patients with IBS-M (Figure 1).” Authors mentioned Rome IV criteria in Methods. Figure 1 is not needed.*
- **Response:** Thanks for your suggestion, we removed it.

- *“Anti-vinculin and anti-CdtB levels were significantly elevated in IBS patients (1.58 ± 0.496 ng/ml and 2.47 ± 0.60 ng/ml) respectively compared to the control subjects (1.13 ± 0.249 ng/ml and 2.1 ± 0.24 ng/ml) respectively with P = 0.001 for both (Table 2).” Authors need to double check vinculin antibodies unit.*
- **Response:** Thanks for your comments, we check it and methodology was modified.

- *In table 3, the first P-value of the anti-cdtB results was calculated based on what? All the other P-values (P1 to P6) are explained on the text below the table.*
- **Response:** Thanks for your comments, we added it and it was already present in the primary submission.

**Discussion**

- *Overall suggestion: the discussion can be better expanded.*
- **Response: **Thanks, done.

- *“There is an extreme necessity for the utilization of accessible and reliable, low-cost biomarkers in identifying IBS for the low-risk population to reduce the use of colonoscopy”. Low-risk population? What do the authors mean with low risk? Please expand a little bit more why the need of development of a test for IBS.*
- **Response: **Thanks for your comment, we clarified this point.
- *“Previous studies have recognized anti-CdtB and anti-vinculin for use as valuable biomarkers in different IBS patients from healthy controls”. Please rephrase it. It is confusing. I encourage the authors to perform a language review of the manuscript. Some suggestions were already included in my review, but I strongly recommend the authors to perform a in deep language review of the entire manuscript.*
- **Response:** Thanks, Paraphrasing was done 

 

- *“Anti-vinculin and anti-CdtB levels in this study were significantly elevated in patients with IBS-D, a concordance finding with Pimentel et al.,  who reported that anti-CdtB and anti-vinculin distinguished IBS-D from IBD, other organic GI diseases and healthy control. In addition, Bayoumy et al.  reported that anti-vinculin could be an important biomarker for IBS-D diagnosis among Egyptian patients.”*
- **Response:** Thanks, corrected

- *“ Cytolethal distending toxin (use abbreviation) is a virulence factor for bacterial pathogens such as Escherichia coli, Salmonella, Shigella, and Campylobacter jejuni, by causing epithelial barrier breakdown and suppression of the acquired immune response to invading pathogens, resulting in an amplified pro-inflammatory response with consequent persistence of bacterial infection. Development of anti-CdtB antibodies occurs in response to secretion of cytolethal distending toxin (use abbreviation) following infection with bacterial pathogens. Molecular mimicry accounts for the potential cross-reaction between anti-CdtB and vinculin with resultant anti-vinculin autoantibody production leading to injury to interstitial cells of Cajal (ICC) with the development of IBS. Based on the suggestion of an association between the metabolic syndrome and liver affection and IBS, this study group performed liver function tests as a simple evaluation of liver affection. However, liver enzymes were normal in IBS patients' studied group, in contrast to reports by Lee et al.” This entire paragraph should be rewritten, it is confusing and not well constructed.*
- **Response:** Thanks, paraphrasing was done
- *The present findings support the hypothesis that IBS may results from post-infectious bacterial gastroenteritis. Moreover, this hypothesis can be applied to all IBS subgroups as both anti-CdtB and anti-vinculin biomarkers were significantly elevated in IBS-C and IBS-D subgroups, with only anti-vinculin being elevated in IBS-M when compared to healthy control*
- **Response: **Many thanks, corrected
- *“This discrepancy in findings may be attributed to the difference in etiology of different IBS subtypes, as it is hypothesized that most cases of post-infectious IBS manifest as IBS-D or IBS-M, with a minority of patients manifesting as IBS-C.  Another factor may make the microbiome profile difference between IBS patient subgroups; bacterial species producing methane are decreased in IBS-D and IBS-M and increased in IBS-C. Patients included in the present study, particularly those in the IBS-C subgroup, may represent patients who develop IBS following infections associated with their microbiota profile changes. These findings need extensive longitudinal studies to be confirmed.” These statements are very confusing, not sure if that can be used to explain why IBS-C also had increased cdtB and vinculin antibodies. This needs to be better clarified. In addition, methane producers are mostly Archaea, not bacteria. Increases in methanogens also represents microbiome changes.*
- **Response:** Many thanks, deleted.
